# Variability of time series barbell kinematics in elite male weightlifters

**DOI:** 10.3389/fspor.2023.1264280

**Published:** 2023-09-13

**Authors:** Ingo Sandau, Georg Langen, Nico Nitzsche

**Affiliations:** ^1^ Department Strength, Power, and Technical Sports, Institute for Applied Training Science, Leipzig, Germany; ^2^ Department Sports Medicine/Sports Biology, Institute of Human Movement Science, University of Technology Chemnitz, Chemnitz, Germany

**Keywords:** snatch, clean, jerk, reliability, performance

## Abstract

**Introduction:**

Barbell kinematics are an essential aspect of assessing weightlifting performance. This study aimed at analyzing the total variability of time series barbell kinematics during repeated lifts in the snatch and the clean and jerk at submaximal and maximal barbell loads.

**Methods:**

In a test-retest design, seven male weightlifters lifted submaximal [85% planned one-repetition maximum (1RMp)] and maximal (97% 1RMp) loads in the snatch and the clean and jerk during training. Barbell trajectory, vertical velocity, and vertical acceleration were determined using video analysis. Standard error of measurement (SEM), intraclass correlation coefficient (ICC), and smallest real difference (SRD) were used to determine the total variability during the lifts. Hedge's g effect size was used to assess differences in SEM between submaximal and maximal loads.

**Results:**

The main findings indicated that variability—in particular for the barbell velocity—was lower at maximal compared to submaximal barbell loads (g = 0.52–2.93). SEM of time series data showed that variability increased in the snatch and the clean and jerk from the 1st pull/dip to the catch position irrespectively of the barbell load.

**Discussion:**

This study presents values of total variability of time series barbell kinematics during the snatch, the clean, and the jerk. Further, the SRD can be used to evaluate changes in barbell kinematics in response to training. In addition, when interpreting barbell kinematics, coaches should take into account that the variability of barbell kinematics can vary depending on the exercise and the barbell load.

## 1. Introduction

Weightlifting requires high levels of muscular strength and barbell handling skills to lift maximal barbell loads in the snatch and the clean and jerk (1, 2). Therefore, a major goal of weightlifting training is to improve the necessary physical abilities using competition lifts (i.e., snatch, clean and jerk) and specific assistant exercises (i.e., power snatch, hang clean, snatch pull) (3). During a lift, the barbell and weightlifter form the barbell-lifter system (4). Based on the barbell lifter system, the analysis of barbell kinematics can be used to assess the lifter’s technical mastery and physical performance measures (i.e., barbell power output) (5). In this context, the barbell trajectory, the vertical barbell velocity, and vertical acceleration are among the most important kinematic parameters (1, 6, 7). Determining the effect of training on changes in barbell kinematics of the individual weightlifter is of major interest for coaches during training and competition. To distinguish a “real” training effect from potential “noise” (i.e., variability) during lifts, the typical magnitude of variability of the lifts needs to be known. For this purpose, an intra-session test-retest experiment can be used to estimate the variability of barbell kinematics in weightlifting exercises (8). In a complex movement task like weightlifting, externally measured variability during repeated lifts is the sum (i.e., total variability) of the variability of motor performance itself, the error of the used measurement system (e.g., video recordings, accelerometers) and the variability related to the used test-condition (9). The typical magnitude of the total variability of barbell kinematics during specific exercises (e.g., snatch, clean and jerk) can be used as a reference point to determine “real” intra-individual changes of barbell kinematics in training and competition (10, 11).

The barbell kinematics in weightlifting are most frequently analyzed based on two-dimensional video recordings (12). As recently pointed out by Nagao und Yamashita (13), the video-based marker-less automatic two-dimensional analysis of barbell distance and velocity highly agrees with data from a three-dimensional infrared motion capture system [intraclass correlation coefficient (ICC): 0.971–0.999; systematic bias: −0.001–0.001 m, −0.034–−0.005 m·s^−1^]. In addition, it has been reported that a video-based automatic marker-less two-dimensional barbell tracking software has a high test-retest reliability for barbell distance, velocity, and acceleration if the same videos where analyzed twice [ICC: 0.98–0.99; standard error of measurement (SEM): 0.005 m, 0.01 m·s^−1^, 0.18 m·s^−2^] (12, 14). Further, the mentioned SEMs associated with the automatic video-analysis of the barbell kinematics are constant and do not change in relation to the barbell load used or exercise performed. Therefore, for the same exercise, changes in total variability during repeated lifts or during a single lift (time series data) are mainly caused by changes in the variability of the motor performance.

Until now, only limited amounts of studies have analyzed the variability of barbell kinematics in weightlifting. Furthermore, the analysis of the variability (i.e., reliability) of biomechanical parameters in weightlifting focused on discrete values rather than on time series data. Therefore, this study aimed to assess the intra-session variability of time series barbell kinematics in weightlifting via reliability-metrics (i.e., SEM, ICC) during maximal and submaximal loads in the snatch and the clean and jerk. We hypothesized that barbell load (submaximal vs. maximal) and lifting phase would affect the variability of the competitive lifts (15, 16).

## 2. Materials and methods

### 2.1. Subjects

Seven elite male weightlifters from the German national team (age: 24.5 ± 4.1 years; body mass: 92.4 ± 23.9 kg, 1RM snatch: 146.4 ± 19.9 kg, 1RM clean and jerk: 179.7 ± 20.3 kg) volunteered to participate in this study. All weightlifters were full time professionals and had a training background of systematic training ranging from 8 to 15, years. Further, all athletes competed regularly on an international level (European and World Championships). They were free from any musculoskeletal or neurological diseases or injuries at the time of data acquisition. The study was conducted according to the latest version of the Declaration of Helsinki and the protocol was approved by the ethical review board of the Institute for Applied Training Science (approval number: ER_2021.22.09_12).

### 2.2. Procedures

This experimental study used an intra-session test-retest design to estimate the trial-to-trial variability of time series based parameters of barbell kinematics in the snatch and the clean and jerk at submaximal and maximal barbell loads in elite male weightlifters. The data were collected during a regular training session during the preparation phase of a macrocycle. Before the tests, an individualized warm-up program was conducted for 15–20 min including cycling on an ergometer at submaximal intensity and mobility exercises with and without the barbell. After the warm-up, weightlifters performed one-repetition maximum (1RM) tests in the snatch, followed by the clean and jerk. During the tests, weightlifters lifted loads starting at approximately 50% of the planned 1RM (1RMp) (=100%) using 8–10 load stages with 1–2 repetitions (12–15 repetitions in total). During the regular training of weightlifters, barbell loads ≥85% of 1RM are commonly used to start with technical skill training (17). Therefore, 85% of 1RMp was used as the minimum (i.e., submaximal) load for the test-retest lifts in the snatch and the clean and jerk, respectively. The maximal load for the test-retest lifts corresponded to ≈97% of the 1RMp for each exercise. The test-retest lifts at 85% and 97% for the snatch and the clean and jerk consisted of two consecutive repetitions each, separated by 2 min of rest. Due to the observational character of this study, daily training routines were not disturbed. Variability analyses were performed for the barbell trajectory, vertical barbell velocity, and vertical barbell acceleration.

All lifts in the snatch and the clean and jerk were video recorded and analyzed using custom-made real-time barbell tracking software (Realanalyzer, IAT, Leipzig, Germany) (12). This software was specifically developed to analyze barbell kinematics during the Olympic lifts in training and competition. Due to the automatic analyze procedure, the weightlifting specific data output, and the attached database, an easy-to-administer measurement system was designed. As mentioned previously, the reliability and validity of the Realanalyzer barbell tracking software is excellent (12). For this investigation, the position of the digital camera (Canon, Legria HF G26) followed a routine set-up. The camera was placed at a distance of 5 m and 1.5 m above the lifting platform, almost perpendicular to the plane of lifting. The Realanalyzer software automatically tracks the barbell during the lift in a video at 50 frames per second with an OpenCV template-matching algorithm. From the automatic tracking, the raw pixel data were smoothed with a cubic spline function. The smoothed pixel data represent the horizontal (x) and vertical (y) position data of the barbell (i.e., barbell trajectory). The vertical barbell velocity (vy) and acceleration (ay) were computed as the 1st and 2nd derivatives of the vertical position data derived from the cubic spline function. Finally, the real position, velocity, and acceleration data were computed via 2D image calibration (diameter of barbell plate).

**Figure 1:**
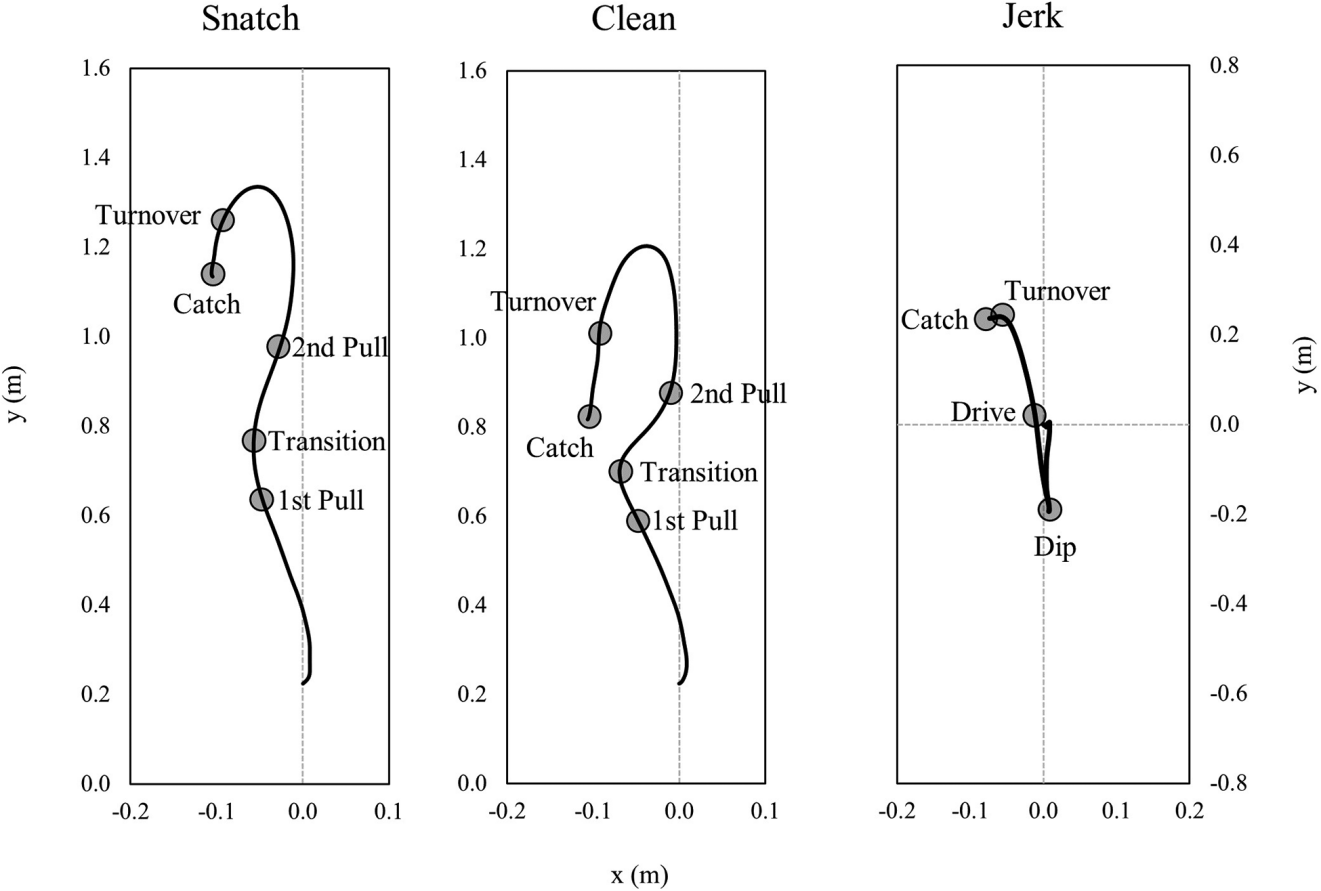
Representative barbell trajectories for the right barbell side for the snatch, the clean, and the jerk with parameters defining the endpoints of the analyzed lifting phases that were used for analyses.

The tracked barbell kinematics were stored as time series data. For the test-retest analysis, time series based barbell parameters were used. Representative examples of the horizontal (x) and vertical (y) barbell position data representing the barbell trajectory during the snatch and the clean and jerk are shown in Figure 1.

**Figure 2:**
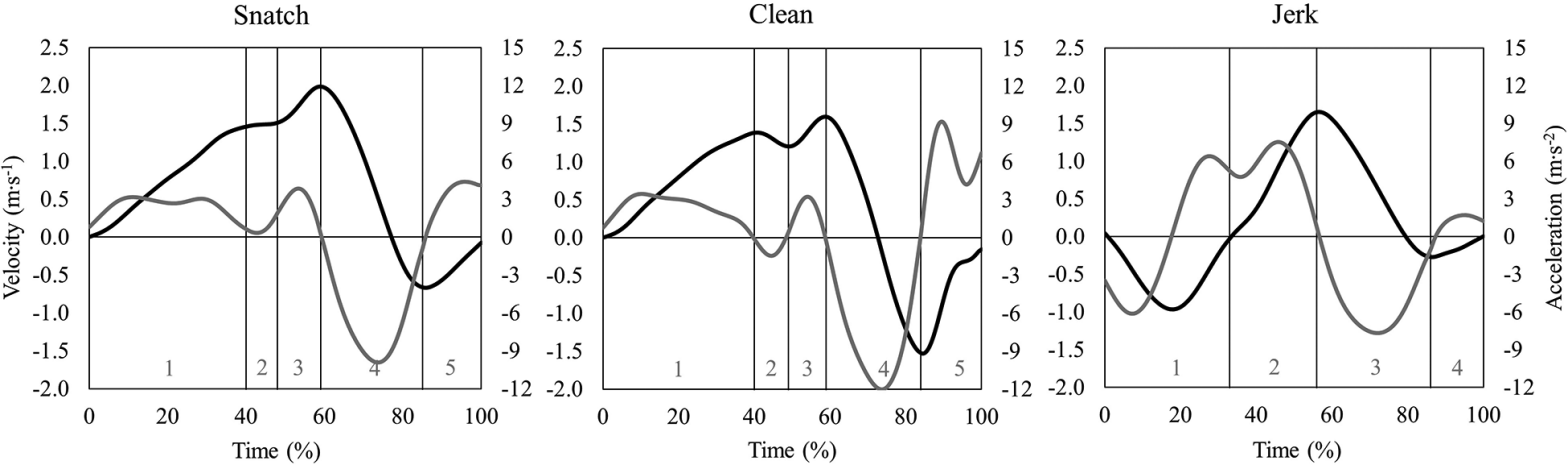
Representative time series data of vertical barbell velocity (black line) and acceleration (grey line) in the snatch, the clean, and the jerk at a maximal barbell load. Lifting phases for the snatch and the clean are coded as: 1st pull (1), transition (2), 2nd pull (3), turnover (4), catch (5). Lifting phases for the jerk are coded as: dip (1), drive (2), turnover (3), catch (4).

Alongside barbell trajectory, time series data of vertical barbell velocity (vy) and vertical barbell acceleration (ay) were used for further analyses (Figure 2).

All lifts were separated into lifting phases, based on barbell kinematics and the athletes' positions during the lifts (18). The phases of the snatch and the clean were: 1st pull (lift-off to the first maximum of knee extension), transition (end of first maximum knee extension to first minimum of knee flexion), 2nd pull [end of first minimum of knee flexion to maximal vertical barbell velocity (v_max_)], turnover [v_max_ to maximal vertical barbell drop velocity (v_min_)] and catch (v_min_ to deep squat position). The phases of the jerk were: dip (upright starting position to lower turning point of the barbell), drive (lower turning point of the barbell to v_max_), turnover (v_max_ to v_min_), and catch (v_min_ to split jerk position). These aforementioned phases were used to temporally align (0–100%) all time series waveforms of barbell position, velocity, and acceleration using piecewise linear length normalization (19). In detail, first, the average percentage time of each lifting phase (i.e., consensus times) was determined in relation to the entire lifting duration. Second, individual time series data was then aligned to these consensus times using linear interpolation (20). With this normalization technique, phases of the lifts were aligned at defined consensus time points for all attempts to eliminate temporal differences of time series waveforms. The aligned and normalized kinematic time series data were used for the statistical analyses.

### 2.3. Statistical analyses

To estimate the trial-to-trial variability, reliability metrics were used (21). First, absolute (SEM) and relative (ICC_2.1_; two-way random, single measures, absolute agreement) reliability indices were calculated for every discrete data point of the time-normalized kinematic time series data (x, y, vy, ay) for the snatch, the clean, and the jerk for submaximal and maximal lifts (22). According to Koo und Li (23), the ICC can be categorized as poor (ICC ≤0.5), moderate (ICC ≤ 0.75), good (ICC ≤ 0.9), and excellent (ICC > 0.9). The SEM for every discrete time point of the normalized kinematic time series data was calculated as proposed by Hopkins (24):

(1) $$\mathrm{SE}M_{i}=\frac{SD_{\mathrm{diff}\_i}}{\sqrt{2}},$$

where SD_diff_ is the sample standard deviation of the test-retest difference scores for every discrete data point (i). From the SEM, the smallest real difference (SRD) (25) for every discrete data point was calculated as:

(2) $$\mathrm{SR}D_{i}=1.96\times \mathrm{SE}M_{i}\times \sqrt{2}.$$

According to equation (2), SRD represents an 95% error interval of the observed differences. In practice, if the difference between two performance measurements ± SRD did not contain zero, we could be 95% confident that the change in the athlete's performance is larger/smaller than a difference caused only by measurement error of this test-retest protocol. Therefore, a “real” difference beyond the measurement error can be assumed (10).

Second, integrated pointwise indices (IPI) were calculated for ICC_2.1_, SEM, and SRD time series data, and for each single lifting phase for the snatch/clean (i.e., 1st pull, transition, 2nd pull, turnover, catch) and the jerk (i.e., dip, drive, turnover, catch) for submaximal and maximal barbell loads. The IPI summarizes the information of ICC_2.1_, SEM, and SRD time series data within each lifting phase [i.e., number of discrete time steps (n)] into a single value (average) (26):

(3) $$X_{\mathrm{IPI}}=\frac{1}{n}\sum_{i=1}^{n}X_{i},$$

where X represents the wildcard for the three aforementioned statistical indices.

In addition to IPI, the variation of IPI for the analyzed period [i.e., sample standard deviation (SD)] was calculated as:

(4) $$SD_{X_{\mathrm{IPI}}}=\sqrt{\frac{1}{n-1}\sum_{i=1}^{n}(X_{i}-X_{\mathrm{IPI}}{)}^{2}.}$$

Third, to analyze differences in SEM_IPI_ between submaximal and maximal loads in the snatch, and the clean and jerk, effect size with bias correction (i.e., Hedges' *g*) was used (27):

(5) $$g=\frac{\mathrm{SE}M_{\mathrm{IPI}\_\mathrm{submax}}-\mathrm{SE}M_{\mathrm{IPI}\_\max}}{\sqrt{\frac{(n_{\mathrm{submax}}-1){\mathrm{SD}}_{\mathrm{SE}M_{\mathrm{IPI}\_\mathrm{submax}}}^{2}+(n_{\max}-1){\mathrm{SD}}_{\mathrm{SE}M_{\mathrm{IPI}\_\max}}^{2}}{n_{\mathrm{submax}}+n_{\max}-2}}}\times \left(1-\frac{3}{4(n_{\mathrm{submax}}+n_{\max})-9}\right).$$

The effect size was interpreted using the conventions outlined by Hopkins (28) as small (*g* > |0.2|), moderate (g > |0.6|), large (g > |1.2|), very large (g > |2.0|), or extremely large (g > |4.0|). An effect size < |0.2| was deemed trivial. Since all time series data of SEM_IPI_ are highly autocorrelated, the analyses of differences between submaximal and maximal loads only rely on point estimates of the effect size without inference statistics to avoid inflating the probability of a type-1 error (false rejection) relative to its declared value. In contrast, standardized mean differences (e.g., Hedges' *g*) are assumed to be not influenced by autocorrelation (29). All statistical analyses were done in R (30) and Microsoft Excel 2016 (Microsoft Corp., Redmond, WA, USA).

## 3. Results

In terms of practical use, total variability should be expressed as a metric of the measured scale (i.e., SEM) to have a direct interpretation of the amount of precision of individual test scores (11). Therefore, we focus on the SEM in the written presentation of the results.

From the results, it can be concluded that lifting phase (Tables 1–3) and barbell load (submaximal vs. maximal, Table 4) influence SEM_IPI_, ICC_IPI_, and consequently SRD_IPI_ in the snatch, the clean, and the jerk.

**Table 1:** Integrated pointwise indices of standard error of measurement (SEM_IPI_ ± one standard deviation), smallest real difference (SRD_IPI_ ± one standard deviation), and intraclass-correlation coefficient (ICC_IPI_ ± one standard deviation) at submaximal and maximal loads of planned one-repetition maximum (1RMp) for the snatch.

			Submax (85% 1RMp)				Max (97% 1RMp)			
			x [m]	y [m]	vy [m·s^−1^]	ay [m·s^−2^]	x [m]	y [m]	vy [m·s^−1^]	ay [m·s^−2^]
Snatch	SEM_IPI_	1st Pull	0.005 ± 0.002	0.007 ± 0.004	0.036 ± 0.01	0.264 ± 0.081	0.004 ± 0.003	0.017 ± 0.01	0.058 ± 0.026	0.332 ± 0.198
		Transition	0.009 ± 0.001	0.015 ± 0.002	0.059 ± 0.008	0.351 ± 0.033	0.005 ± 0.001	0.022 ± 0.002	0.031 ± 0.017	0.311 ± 0.033
		2nd Pull	0.011 ± 0.001	0.016 ± 0.002	0.05 ± 0.011	0.398 ± 0.085	0.009 ± 0.002	0.019 ± 0.003	0.041 ± 0.017	0.26 ± 0.054
		Turnover	0.014 ± 0.003	0.012 ± 0.003	0.062 ± 0.024	0.679 ± 0.243	0.013 ± 0.002	0.011 ± 0.004	0.035 ± 0.012	0.323 ± 0.171
		Catch	0.023 ± 0.003	0.017 ± 0.001	0.055 ± 0.013	0.722 ± 0.238	0.021 ± 0.002	0.011 ± 0.003	0.025 ± 0.006	0.436 ± 0.091
	SRD_IPI_	1st Pull	0.014 ± 0.007	0.019 ± 0.011	0.099 ± 0.028	0.733 ± 0.225	0.012 ± 0.008	0.047 ± 0.028	0.16 ± 0.028	0.921 ± 0.225
		Transition	0.025 ± 0.001	0.043 ± 0.006	0.163 ± 0.022	0.972 ± 0.091	0.015 ± 0.002	0.06 ± 0.007	0.085 ± 0.022	0.86 ± 0.091
		2nd Pull	0.029 ± 0.003	0.045 ± 0.005	0.139 ± 0.03	1.102 ± 0.237	0.024 ± 0.005	0.053 ± 0.007	0.112 ± 0.03	0.721 ± 0.237
		Turnover	0.039 ± 0.007	0.034 ± 0.009	0.173 ± 0.066	1.881 ± 0.674	0.036 ± 0.007	0.032 ± 0.011	0.098 ± 0.066	0.894 ± 0.674
		Catch	0.064 ± 0.008	0.048 ± 0.001	0.154 ± 0.037	2.001 ± 0.659	0.058 ± 0.006	0.031 ± 0.008	0.071 ± 0.037	1.208 ± 0.659
	ICC_IPI_	1st Pull	0.76 ± 0.17	0.67 ± 0.29	0.77 ± 0.19	0.81 ± 0.14	0.96 ± 0.06	0.49 ± 0.23	0.66 ± 0.22	0.74 ± 0.21
		Transition	0.87 ± 0.02	0.89 ± 0.03	0.85 ± 0.02	0.81 ± 0.03	0.99 ± 0.00	0.82 ± 0.02	0.91 ± 0.08	0.83 ± 0.03
		2nd Pull	0.91 ± 0.02	0.92 ± 0.04	0.85 ± 0.01	0.82 ± 0.08	0.98 ± 0.00	0.88 ± 0.04	0.78 ± 0.04	0.86 ± 0.10
		Turnover	0.91 ± 0.03	0.98 ± 0.01	0.65 ± 0.27	0.33 ± 0.37	0.97 ± 0.00	0.96 ± 0.03	0.78 ± 0.14	0.47 ± 0.25
		Catch	0.82 ± 0.04	0.97 ± 0.01	0.46 ± 0.27	0.45 ± 0.35	0.95 ± 0.00	0.98 ± 0.01	0.49 ± 0.41	0.44 ± 0.28

**Table 2:** Integrated pointwise indices of standard error of measurement (SEM_IPI_ ± one standard deviation), smallest real difference (SRD_IPI_ ± one standard deviation), and intraclass-correlation coefficient (ICC_IPI_ ± one standard deviation) at submaximal and maximal loads of planned one-repetition maximum (1RMp) for the clean.

			Submax (85% 1RMp)				Max (97% 1RMp)			
			x [m]	y [m]	vy [m·s^−1^]	ay [m·s^−2^]	x [m]	y [m]	vy [m·s^−1^]	ay [m·s^−2^]
Clean	SEM_IPI_	1st Pull	0.006 ± 0.002	0.01 ± 0.005	0.055 ± 0.029	0.343 ± 0.273	0.006 ± 0.003	0.013 ± 0.009	0.043 ± 0.014	0.256 ± 0.076
		Transition	0.008 ± 0.001	0.014 ± 0.003	0.02 ± 0.004	0.241 ± 0.07	0.01 ± 0.002	0.024 ± 0.002	0.033 ± 0.004	0.266 ± 0.155
		2nd Pull	0.011 ± 0.001	0.011 ± 0.004	0.039 ± 0.005	0.29 ± 0.088	0.011 ± 0.002	0.019 ± 0.002	0.025 ± 0.004	0.353 ± 0.133
		Turnover	0.011 ± 0.002	0.023 ± 0.013	0.15 ± 0.076	1.159 ± 0.853	0.011 ± 0.002	0.014 ± 0.002	0.044 ± 0.016	0.38 ± 0.195
		Catch	0.015 ± 0.003	0.028 ± 0.011	0.072 ± 0.016	0.829 ± 0.203	0.016 ± 0.002	0.013 ± 0.001	0.082 ± 0.03	1.003 ± 0.172
	SRD_IPI_	1st Pull	0.016 ± 0.006	0.026 ± 0.012	0.153 ± 0.08	0.95 ± 0.755	0.016 ± 0.007	0.035 ± 0.024	0.118 ± 0.038	0.708 ± 0.209
		Transition	0.022 ± 0.002	0.038 ± 0.007	0.055 ± 0.01	0.667 ± 0.193	0.026 ± 0.003	0.065 ± 0.004	0.09 ± 0.01	0.736 ± 0.428
		2nd Pull	0.03 ± 0.002	0.031 ± 0.01	0.106 ± 0.014	0.802 ± 0.243	0.029 ± 0.005	0.051 ± 0.005	0.068 ± 0.011	0.977 ± 0.368
		Turnover	0.03 ± 0.004	0.063 ± 0.036	0.413 ± 0.211	3.209 ± 2.361	0.028 ± 0.005	0.037 ± 0.005	0.121 ± 0.045	1.052 ± 0.539
		Catch	0.041 ± 0.008	0.078 ± 0.029	0.198 ± 0.044	2.294 ± 0.561	0.043 ± 0.005	0.034 ± 0.002	0.227 ± 0.081	2.777 ± 0.476
	ICC_IPI_	1st Pull	0.79 ± 0.13	0.34 ± 0.41	0.53 ± 0.43	0.63 ± 0.28	0.77 ± 0.11	0.60 ± 0.08	0.77 ± 0.10	0.77 ± 0.19
		Transition	0.91 ± 0.02	0.94 ± 0.02	0.85 ± 0.06	0.88 ± 0.03	0.91 ± 0.01	0.77 ± 0.08	0.94 ± 0.00	0.76 ± 0.28
		2nd Pull	0.88 ± 0.04	0.95 ± 0.02	0.63 ± 0.19	0.91 ± 0.12	0.93 ± 0.03	0.89 ± 0.01	0.94 ± 0.02	0.70 ± 0.37
		Turnover	0.91 ± 0.02	0.86 ± 0.09	0.03 ± 0.55	0.17 ± 0.44	0.93 ± 0.02	0.95 ± 0.01	0.75 ± 0.11	0.72 ± 0.21
		Catch	0.81 ± 0.04	0.84 ± 0.09	0.56 ± 0.29	0.50 ± 0.24	0.89 ± 0.01	0.95 ± 0.01	0.65 ± 0.27	0.68 ± 0.15

**Table 3:** Integrated pointwise indices of standard error of measurement (SEM_IPI_ ± one standard deviation), smallest real difference (SRD_IPI_ ± one standard deviation), and intraclass-correlation coefficient (ICC_IPI_ ± one standard deviation) at submaximal and maximal loads of planned one-repetition maximum (1RMp) for the jerk.

			Submax (85% 1RMp)				Max (97% 1RMp)			
			x [m]	y [m]	vy [m·s^−1^]	ay [m·s^−2^]	x [m]	y [m]	vy [m·s^−1^]	ay [m·s^−2^]
Jerk	SEM_IPI_	Dip	0.007 ± 0.002	0.012 ± 0.006	0.068 ± 0.025	0.765 ± 0.334	0.007 ± 0.002	0.008 ± 0.004	0.031 ± 0.008	0.278 ± 0.063
		Drive	0.013 ± 0.002	0.011 ± 0.002	0.051 ± 0.014	0.685 ± 0.093	0.009 ± 0.001	0.012 ± 0.002	0.037 ± 0.01	0.657 ± 0.188
		Turnover	0.016 ± 0.001	0.015 ± 0.002	0.059 ± 0.012	0.434 ± 0.196	0.013 ± 0.003	0.009 ± 0.003	0.059 ± 0.006	0.358 ± 0.18
		Catch	0.018 ± 0.001	0.021 ± 0.001	0.037 ± 0.017	0.572 ± 0.095	0.024 ± 0.004	0.018 ± 0.004	0.058 ± 0.004	0.875 ± 0.349
	SRD_IPI_	Dip	0.019 ± 0.006	0.033 ± 0.017	0.186 ± 0.068	2.118 ± 0.925	0.02 ± 0.004	0.021 ± 0.009	0.085 ± 0.02	0.77 ± 0.174
		Drive	0.035 ± 0.005	0.029 ± 0.005	0.139 ± 0.039	1.895 ± 0.257	0.023 ± 0.001	0.032 ± 0.005	0.102 ± 0.026	1.818 ± 0.52
		Turnover	0.045 ± 0.003	0.042 ± 0.006	0.162 ± 0.033	1.202 ± 0.541	0.036 ± 0.008	0.024 ± 0.006	0.162 ± 0.015	0.991 ± 0.497
		Catch	0.049 ± 0.002	0.057 ± 0.002	0.102 ± 0.045	1.583 ± 0.262	0.064 ± 0.011	0.05 ± 0.009	0.161 ± 0.011	2.423 ± 0.967
	ICC_IPI_	Dip	0.31 ± 0.13	0.22 ± 0.17	0.42 ± 0.23	0.49 ± 0.27	0.57 ± 0.18	0.69 ± 0.16	0.82 ± 0.13	0.93 ± 0.03
		Drive	0.01 ± 0.05	0.58 ± 0.13	0.61 ± 0.33	0.59 ± 0.21	0.87 ± 0.01	0.38 ± 0.31	0.83 ± 0.08	0.46 ± 0.42
		Turnover	0.45 ± 0.15	0.62 ± 0.12	0.49 ± 0.24	0.68 ± 0.19	0.87 ± 0.00	0.79 ± 0.16	0.77 ± 0.03	0.70 ± 0.31
		Catch	0.61 ± 0.01	0.38 ± 0.04	0.55 ± 0.25	0.50 ± 0.27	0.84 ± 0.01	0.84 ± 0.03	0.51 ± 0.25	0.55 ± 0.11

**Table 4:** Hedge's g effect size measures for differences of integrated pointwise indices of standard error of measurement between submaximal (85%) and maximal (97%) barbell loads of the planned one-repetition maximum for the snatch and the clean and jerk.

	Lifting Phase	x	y	vy	ay
Snatch	1st Pull	0.26	−1.30	−1.11	−0.44
	Transition	7.26	−2.81	2.03	1.17
	2nd Pull	1.13	−1.16	0.66	1.86
	Turnover	0.46	0.26	1.42	1.66
	Catch	0.98	3.44	2.85	1.56
Clean	1st Pull	−0.06	−0.46	0.52	0.41
	Transition	−1.55	−4.72	−3.63	−0.20
	2nd Pull	0.28	−2.64	2.93	−0.53
	Turnover	0.42	0.98	1.80	1.18
	Catch	−0.45	2.08	−0.42	−0.87
Jerk	Dip	−0.21	0.86	1.90	1.90
	Drive	3.71	−0.71	1.07	0.18
	Turnover	1.42	3.00	0.02	0.38
	Catch	−1.92	1.11	−1.70	−1.11

x, horizontal barbell distance; y, vertical barbell distance; vy, vertical barbell velocity; ay, vertical barbell acceleration.

In general, the barbell load and lifting phase less substantially affect the SEM_IPI_ of the horizontal barbell trajectory in the snatch and the jerk, as opposed to the clean (Table 4). However, for the vertical barbell trajectory in the snatch and clean, the 1st pull, transition, and 2nd pull display smaller SEM_IPI_ at submaximal loads. In contrast, the SEM_IPI_ of vertical trajectory in the jerk is less at maximal loads. The vertical barbell velocity displays smaller SEM_IPI_ at maximal loads for the snatch (except 1st pull), the clean (except transition), and the jerk (except turnover and catch). Finally, in the snatch, the vertical barbell acceleration displays smaller SEM_IPI_ at maximal loads (except 1st pull). In contrast, the SEM_IPI_ of vertical barbell acceleration in the jerk is less during the dip at maximal loads and less during the catch at submaximal loads, respectively.

**Figure 3:**
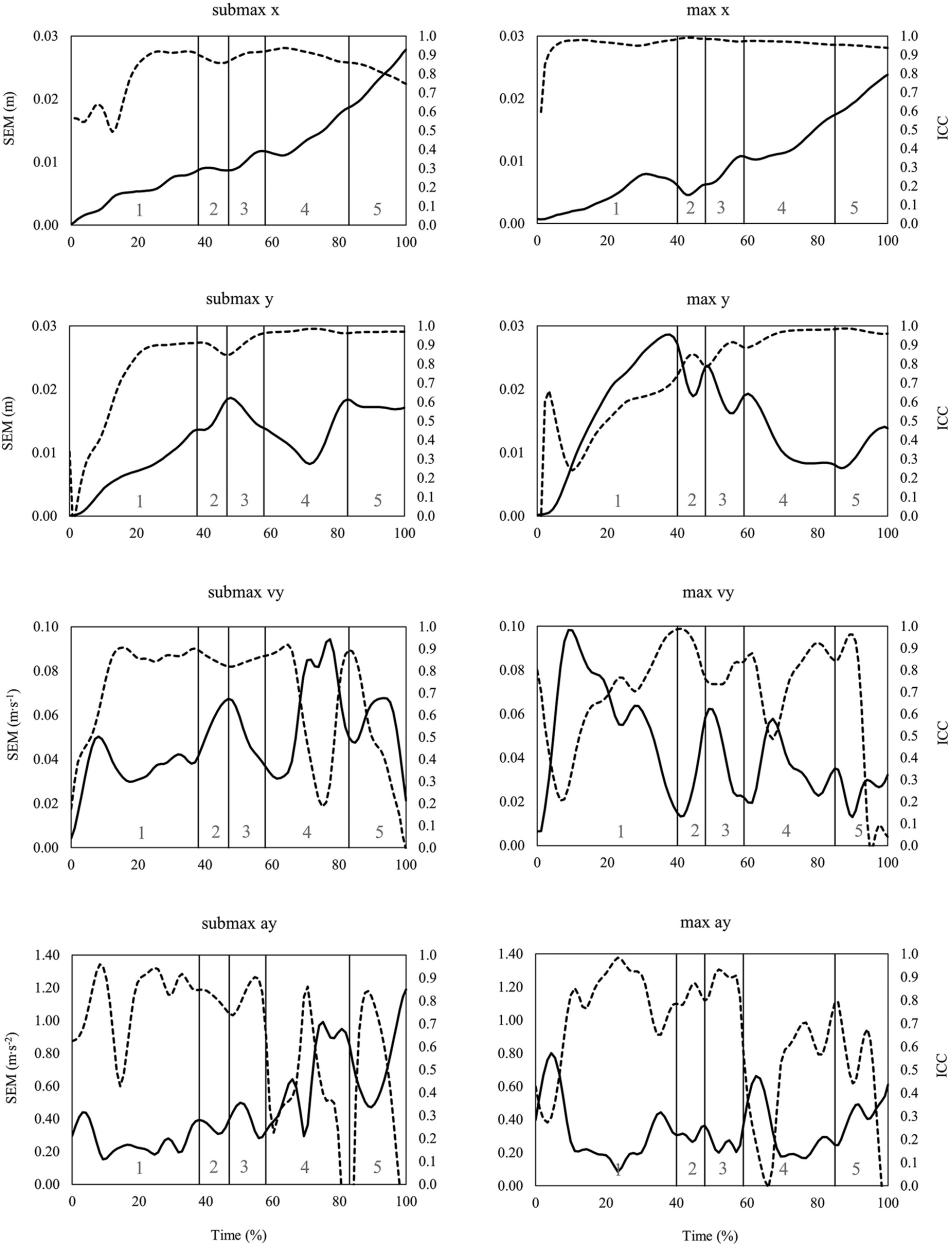
Time series data of standard error of measurement (SEM, solid line) and intraclass correlation coefficient (ICC, dashed line) for the horizontal (x) and vertical (y) barbell trajectory, vertical barbell velocity (vy) and vertical barbell acceleration (ay) during the snatch at submaximal (left) and maximal (right) barbell loads. Lifting phases are coded as: 1st pull (1), transition (2), 2nd pull (3), turnover (4), catch (5).

**Figure 4:**
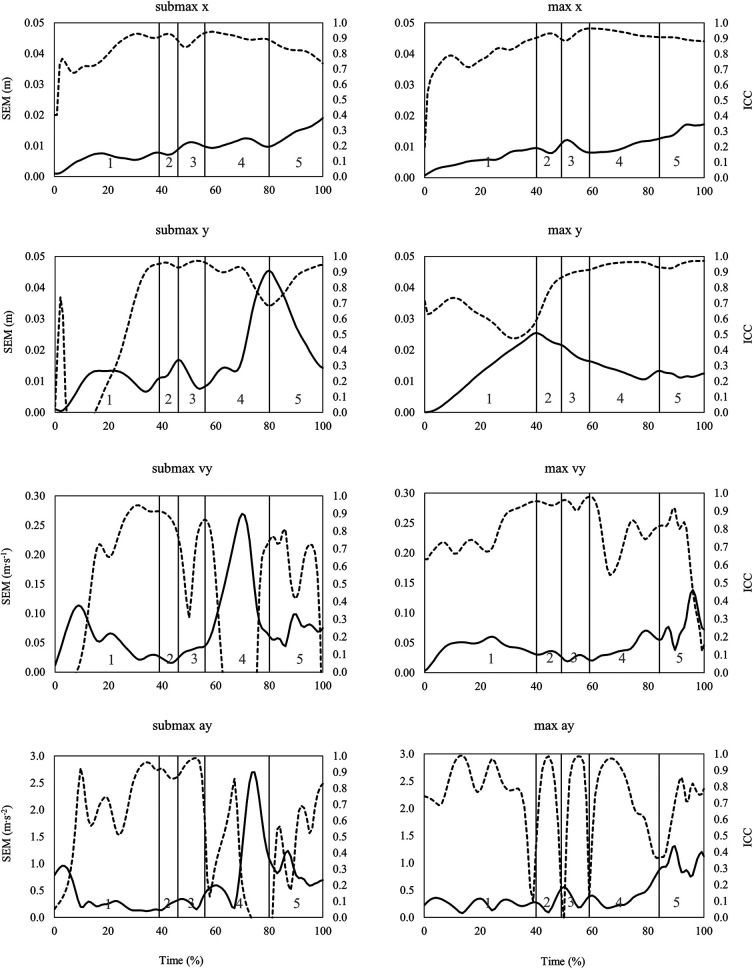
Time series data of standard error of measurement (SEM, solid line) and intraclass correlation coefficient (ICC, dashed line) for the horizontal (x) and vertical (y) barbell trajectory, vertical barbell velocity (vy) and vertical barbell acceleration (ay) during the clean at submaximal (left) and maximal (right) barbell loads. Lifting phases are coded as: 1st pull (1), transition (2), 2nd pull (3), turnover (4), catch (5).

**Figure 5:**
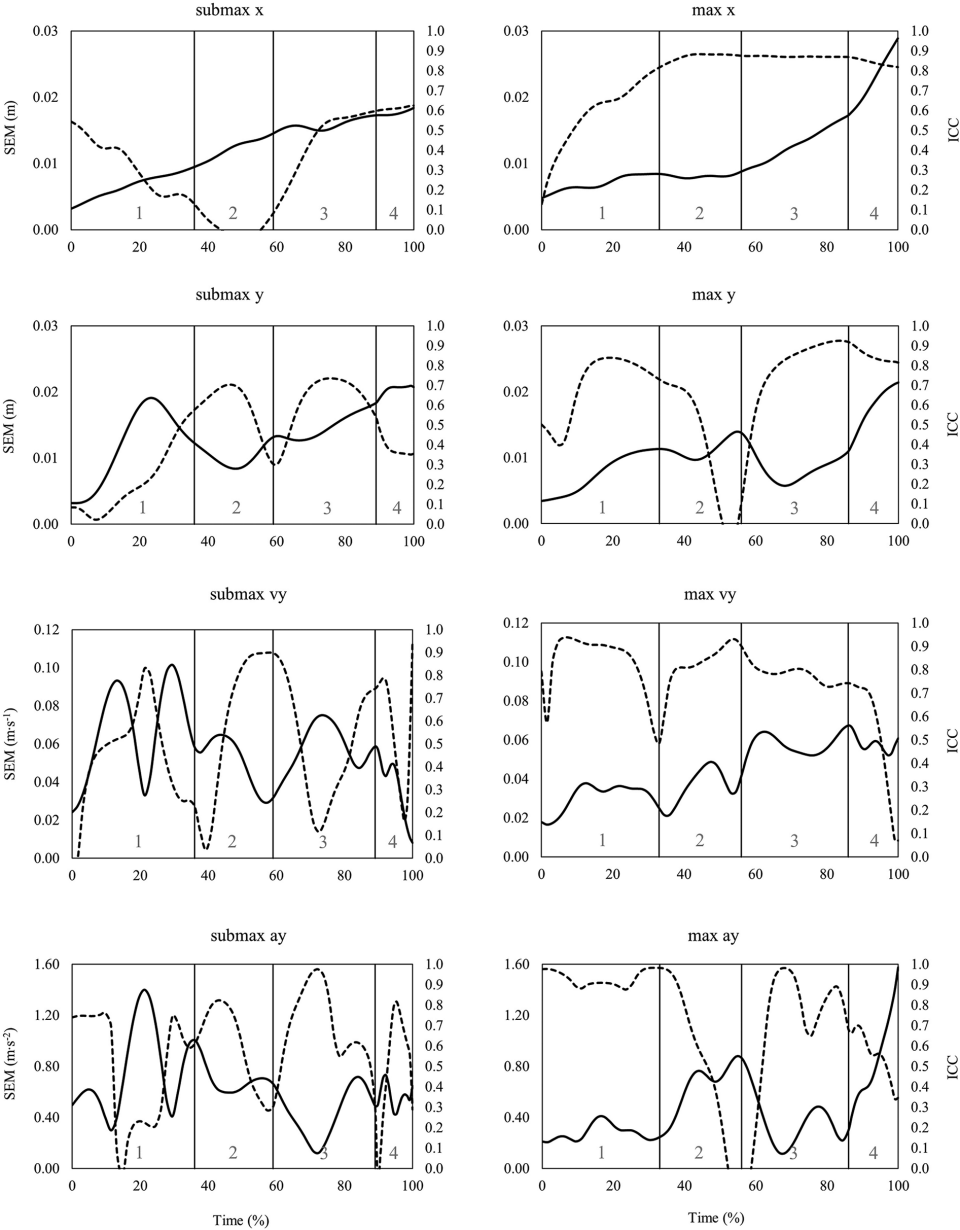
Time series data of standard error of measurement (SEM, solid line) and intraclass correlation coefficient (ICC, dashed line) for the horizontal (x) and vertical (y) barbell trajectory, vertical barbell velocity (vy) and vertical barbell acceleration (ay) during the jerk at submaximal (left) and maximal (right) barbell loads. Lifting phases are coded as: dip (1), drive (2), turnover (3), catch (4).

When looking at the time series data of SEM and ICC across the snatch, the clean, and the jerk (Figures 3–5), it can be summarized that the variability of barbell trajectory (x and y), vertical barbell velocity, and vertical barbell acceleration tend to increase as the lift progresses. In addition, it is worth pointing out that during the lift in the snatch and the clean, the variability of vertical barbell velocity is smaller (i.e., smaller SEM) at the end of the single lifting phases than within the lifting phases (except transition).

## 4. Discussion

This study aimed to analyze the intra-session total variability, in terms of absolute (SEM) and relative (ICC) reliability, of barbell kinematics in elite weightlifters during repeated lifts in the snatch, the clean, and the jerk at submaximal (85% 1RMp) and maximal loads (97% 1RMp). Concurring with our hypothesis, the total variability during the snatch, the clean, and the jerk depends on the barbell load and lifting phase. In general, variability in the snatch (primarily in barbell velocity) decreased at maximal compared to submaximal barbell loads. In addition, total variability of time series kinematics increased at both loads as the lifts in the snatch and the clean and jerk progressed.

In the training of elite weightlifters, objectifying specific barbell handling skills using biomechanical analyses (e.g., video analysis) is an important factor to control and monitor lifting performance (12). Accordingly, attention should be given to the inherent total variability of biomechanical data for a specific test (9). In this context, the total variability of maximal barbell velocity during the power clean (40–100% of 1RM) in competitive weightlifters was previously analyzed, showing an SEM ranging from 0.05–0.09 m·s^−1^ measured with a linear position transducer (16). In comparison, the assessed total variability (i.e., SEM_IPI_) in our study for vertical barbell velocity time series data of the snatch, clean, and jerk is slightly smaller (majority of SEM_IPI_ = 0.01–0.06 m·s^−1^). Additionally, for total variability of barbell acceleration in the power snatch, power clean and jerk in strength-trained athletes, the SEM has previously been reported to be 1.77 m·s^−2^, 1.0 m·s^−2^, and 0.55 m·s^−2^, respectively, using an accelerometer attached to the barbell (31). Again, in our study, the total variability of vertical barbell acceleration time series data in the three exercises is slightly smaller (SEM_IPI_ = 0.26–1.0 m·s^−2^). The aforementioned differences in SEM can be explained by the technical mastery of the elite weightlifters in our study and the high precision of the used barbell tracking software. Although barbell trajectory is of major interest in weightlifting research (6, 32), to our knowledge, no studies have assessed total variability (i.e., SEM) of barbell trajectory in the snatch, the clean, and the jerk. Therefore, our study outcomes may give additional insights into how to interpret individual changes in barbell trajectory during training and competition. Furthermore, based on the smaller SEM_IPI_ at maximal compared to submaximal barbell loads, future studies on variability assessment in weightlifting and strength training should account for barbell load as an additional component that can moderate the total variability of movement outcomes.

Typically, reliability indices of biomechanical variables often rely on single points/events (i.e., discrete parameters) from an entire time series of the measured data. As previously presented for cycling (33) and one-leg hops (26), reporting and analyzing reliability indices as time series data (waveforms or aggregated as integrated pointwise indices), rather than as discrete points, provide more detailed insight into changes in variability over time. In the case of barbell kinematics in the snatch, the clean and the jerk, calculating the reliability indices as time series showed that total variability (i.e., SEM, SRD) increased in later stages of the lift, in particular for the horizontal barbell trajectory. For example, the SRD_IPI_ for horizontal barbell trajectory in the snatch is about 4 cm in the turnover phase and 6 cm in the catch phase (Table 1). Furthermore, time series variability waveforms can be used to select time points with low variability from which discrete parameters can be derived. In this context, during the 1st pull and 2nd pull of the snatch, the variability of vertical barbell velocity is lowest at the end of the lifting phases. These time points would serve well to define discrete vertical barbell velocity parameters that the coach can use to determine the effects of training on barbell kinematics.

As mentioned previously, the total variability during repeated lifts mainly reflects the variability of the motor performance (i.e., movement). With ongoing skill development, the movement pattern shows less variable and more stable aspects of the movement (34). In this context, the more stable movement patterns (i.e., less variability) were assumed to be those which are goal-directed (34) and hence related to the movement outcome (i.e., performance). Further, it has been discussed that motor variability is regulated that more reproducible output is aimed at situations with a high “reward” (35). Viewed the other way around, specific parts of a movement with lower trial-to-trial variability might be important concerning the outcome. Transferring this idea to the time series SEM data it can be concluded that during maximal lifts, achieving a desired v_max_ in the snatch, the clean, and the jerk, is one of the “goals” within the lifting movement since variability at these time points is lower compared to others. This fact may underline the importance of the vertical barbell (threshold) velocity to lift a maximal load (18). In contrast, the higher variability in vertical barbell velocity at submaximal barbell loads may be interpreted as less “rewarded” movements with less “precision” needed to achieve the desired goal. Specifically, at submaximal loads, a good lift can be achieved with a wider range of possible v_max_ values. However, at a maximal barbell load only “one solution” of v_max_ exists that is related with a good lift, i.e., the threshold velocity of the barbell at the end of the second pull. However, the above-mentioned theoretical assumptions may be of interest for further research.

In conclusion, guiding the weightlifter’s training process based on kinematic measurements of the barbell can improve weightlifting performance. For practical use, knowledge of the typical total variability (SEM) of kinematic barbell parameters can be useful to detect the smallest “real” difference (SRD) in kinematic barbell parameters during training and competition. Our study presents reference values of SEM/SRD for barbell kinematics (i.e., x, y, vy, ay) of the snatch, the clean, and the jerk at submaximal and maximal barbell loads. These reference values can be used to estimate changes in barbell kinematics in response to training. In this context, during training, coaches should take into account that the variability of barbell kinematics can vary depending on the exercise and the barbell load.

For this study, some limitations need to be acknowledged. First, from research on vertical jumps, it is known that variability in performance changes over time (36). In our study, the intra-session test-retest reliability was assessed during one singular training session during the preparation phase of a macrocycle. Therefore, variability at other time points during a macrocycle may have been higher or lower than reported in our study. Second, our kinematic analyses of the barbell were based on a custom build video tracking software with limited access. Using another device for the kinematic barbell analysis may affect the total variability (e.g., SEM). Third, the presented SEM/SRD is specific to the population under investigation (i.e., elite male weightlifters). Therefore, care should be taken when using these reference values for other populations. Finally, the cohort size was small. However, given that the study was conducted with elite athletes performing on an international level (German national team), the overall population to draw the sample is small. This is a well-known limitation when performing research in elite sports.

## Data Availability

The raw data supporting the conclusions of this article will be made available by the authors, without undue reservation.
